# Localized Biphasic Malignant Peritoneal Mesothelioma with Rhabdoid Features Involving the Liver: Case Report and Review of the Literature

**DOI:** 10.1155/2019/2732674

**Published:** 2019-07-28

**Authors:** Dalal Hassan, Saverio Ligato

**Affiliations:** Department of Pathology and Laboratory Medicine, Hartford Hospital, Hartford, CT, USA

## Abstract

**Introduction:**

Localized malignant mesotheliomas, defined as sharply circumscribed tumors of the serosal membrane with the microscopic appearance of diffuse malignant mesothelioma, are rare tumors; their behavior and prognosis are uncertain. Intrahepatic mesotheliomas are postulated to arise from mesothelial cells of Glisson's capsule.

**Case Presentation:**

A 69-year-old female with no history of asbestos exposure presented with a one-month history of increasing abdominal pain associated with constitutional symptoms. Computerized Tomography (CT) scan of the abdomen and pelvis revealed a sizable soft tissue mass within the right paracolic gutter, abutting the inferior hepatic margin, the lateral abdominal wall, and descending colon. Ultrasound-guided biopsy of the mass suggested a poorly differentiated hepatocellular carcinoma. There was no disease elsewhere on PET scan. Surgical resection of the mass was performed. Pathological assessment suggested the tumor to be arising from the liver with invasion of the liver, abdominal wall musculature, and the adventitial surface of the ascending colon. A final diagnosis of localized biphasic malignant peritoneal mesothelioma with rhabdoid features was rendered based on morphology and the result of immunohistochemical studies. The abdominal wall margin was positive. The patient progressed over the course of 6 months despite receiving adjuvant chemotherapy and immunotherapy with metastases and a decline in performance status and was transitioned to hospice.

**Conclusion:**

Localized malignant peritoneal mesotheliomas are rare tumors that may present clinically as a liver mass and simulate primary hepatic or secondary tumors. Definitive diagnosis is obtained by surgical resection in most cases. The clinical outcome is variable with most cases having a poor outcome.

## 1. Introduction

Malignant mesotheliomas are rare tumors that reportedly account for 0.2% of all malignant tumors. Malignant peritoneal mesotheliomas occurring in the peritoneum have an even lower incidence [1]. Most cases of malignant peritoneal mesothelioma are of the diffuse type, and localized cases are rare. Localized malignant mesotheliomas are defined as sharply circumscribed tumors of the serosal membrane with the microscopic appearance of diffuse malignant mesotheliomas, without any evidence of infiltration. Diffuse malignant mesotheliomas always show gross and/or microscopic evidence of widespread tumor on the serosal surface, in the form of either individual tumor nodules, rind around viscera, or tumor caking [2]. Some authors suggest that diffuse and localized malignant mesotheliomas should be separated, because the later has a localized presentation, better prognosis, and different biological behavior [3]. Primary intrahepatic mesotheliomas are malignant tumors that have been postulated to arise from the mesothelial cell layer covering Glisson's capsule of the liver [4]. We report a case of localized biphasic malignant peritoneal mesothelioma with rhabdoid features involving the liver in a 69-year-old female with no prior history of asbestos exposure with a poor outcome that was suggested to be a poorly differentiated hepatocellular carcinoma on image-guided biopsy. To our knowledge, this is the first case report of localized malignant peritoneal mesothelioma presenting with both biphasic and rhabdoid features. The aim is to increase awareness of localized peritoneal mesothelioma among surgical pathologists, as they can mimic as primary or secondary hepatic tumors clinically and histologically and review the literature with regards to prognosis of these uncommon tumors.

## 2. Case Presentation

A 69-year-old female with a past medical history of coronary artery disease, hypertension, hyperlipidemia, and polymyalgia rheumatica presented with a one-month history of increasing abdominal pain associated with weakness, fatigue, weight loss, and loss of appetite. She had no known history of malignancy nor was she exposed to asbestos in the past.

Laboratory findings revealed a WBC count of 25.8 (reference range: 3.5-12.0 x 109/L) with absolute neutrophil and monocyte counts of 23.05 (reference range: 3000-5800 x 106/L) and 1.67 (reference range: 300-500 x 106/L), a hemoglobin level of 7.7 (reference range for females: 120-160 g/L) with an MCV of 78 (reference range: 76-100 fL), an RDW of 20.7 (reference range: 11.5-14.3%), a platelet count of 729 (reference range: 150-400 x 109/L), a globulin level of 5.3 (reference range: 1.5 - 3.9 g/dL), and a globulin/albumin ratio of 0.4 (reference range: 1.0 - 1.8 ratio).

**Figure 1:**
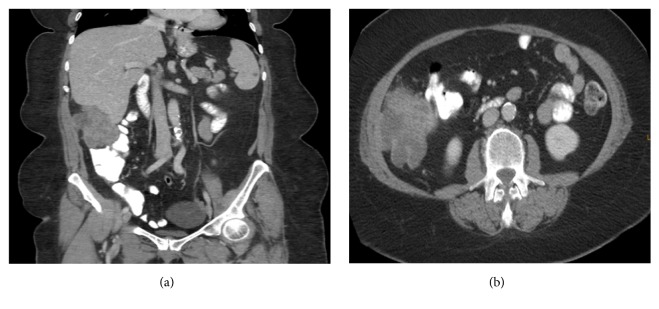
(a, b) CT abdomen/pelvis findings.

Computed Tomography (CT) scan of the abdomen and pelvis showed a soft tissue mass of 9.5 x 8.5 x 7 cm within the right paracolic gutter, abutting the inferior hepatic margin, the lateral abdominal wall, and effacing the descending colon with an extensive solid component and low attenuation, possibly representing necrosis (Figures 1(a) and 1(b)).

Additional findings included a left adnexal cystic mass measuring 8.5 x 6.7 cm without calcifications or a discrete solid wall component. The finding of a left adnexal mass prompted the measurement of serum CA-125, which was within normal limits. The patient underwent a colonoscopy which did not show any intrinsic involvement of the colon.

Ultrasound guided biopsy of the mass suggested a poorly differentiated hepatocellular carcinoma.

A preoperative PET scan showed increased uptake in the mass only, with no evidence of uptake in the left adnexal mass or any other area.

**Figure 2:**
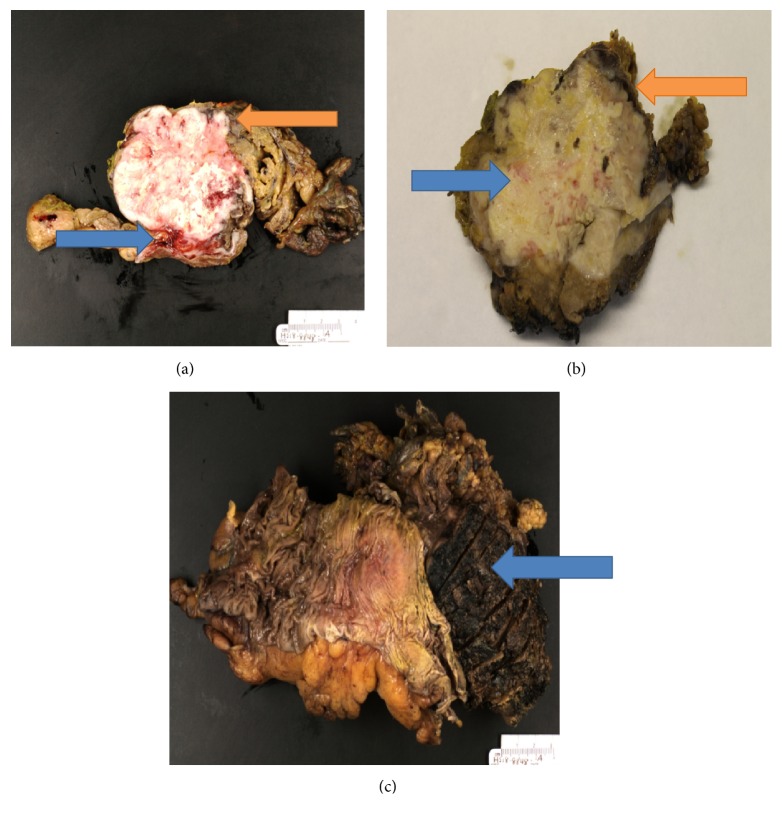
Gross findings: (a) Tan-white necrotic mass abutting the abdominal wall (blue arrow) and infiltrating the omental fat (orange arrow); (b) mass with extensive necrosis abutting the liver (blue arrow) and anterior abdominal wall (orange arrow); (c) tumor abutting the adventitial surface of the ascending colon (blue arrow).

The patient then underwent surgical debulking of the mass for therapeutic purposes and further characterization of the tumor in the form of a laparoscopic assisted wedge resection of segment 6 of liver, right colectomy, and an abdominal wall resection with a partial omentectomy after confirming the absence of metastatic disease on laparoscopy. Gross examination revealed a 9 x 8.0 x 6.0 cm tan-pink, soft, centrally necrotic tumor within the left lobe of the liver, abutting the overlying liver capsule, extending through the capsule into the attached abdominal wall, resulting in retraction of the abdominal wall, and extending into the adjacent omental fat. The tumor also appeared to retract and abut the right colon without grossly extending into the bowel wall (Figures 2(a)–2(c)).

**Figure 3:**
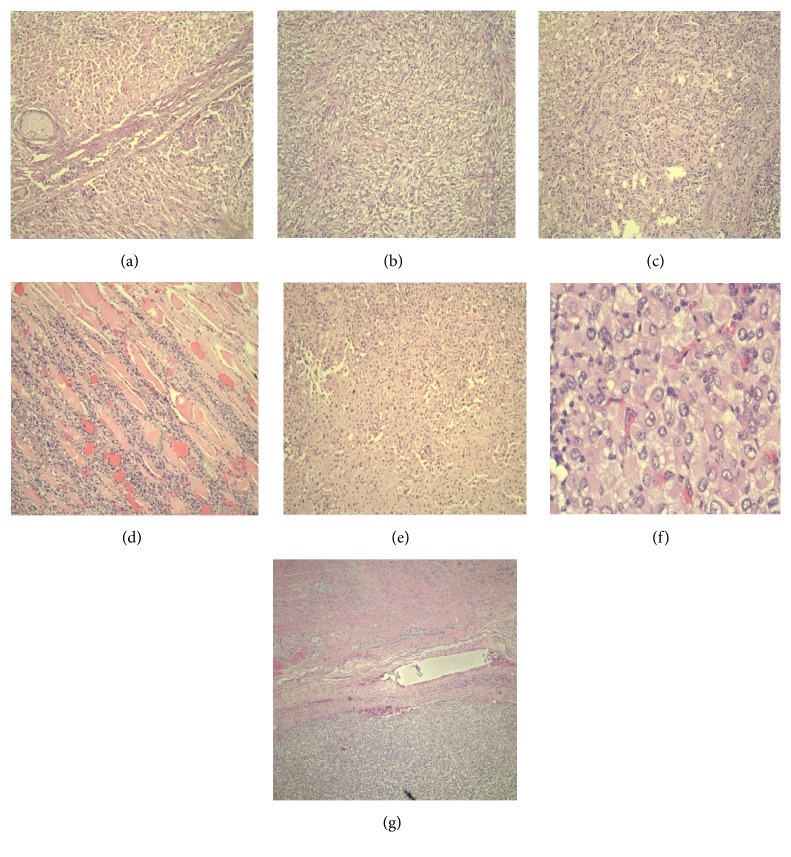
Microscopic findings: (a) tumor arising from surface of liver; (b) spindle cell component; (c) tumor focally invading the liver; (d) tumor invading the abdominal wall musculature; (e) epithelioid component; (f) tumor invading colonic adventitia (g) rhabdoid component.

Microscopically, the tumor was comprised of three components, one component consisting of sheets of pleomorphic polygonal (epithelioid) cells with occasional intracytoplasmic vacuoles intimately associated with fascicles of pleomorphic spindled cells which formed the second component, with interspersed large areas of geographic necrosis. The third component was focal, consisting of cells with eccentrically located nuclei, intranuclear cytoplasmic inclusions, and an abundant amount of glassy, eosinophilic cytoplasm, consistent with rhabdoid cells. The tumor appeared to arise from the surface of the liver and focally invaded the liver and invaded abdominal wall musculature, omental fat, and adventitia of the ascending colon (Figures 3(a)–3(g)).

**Figure 4:**
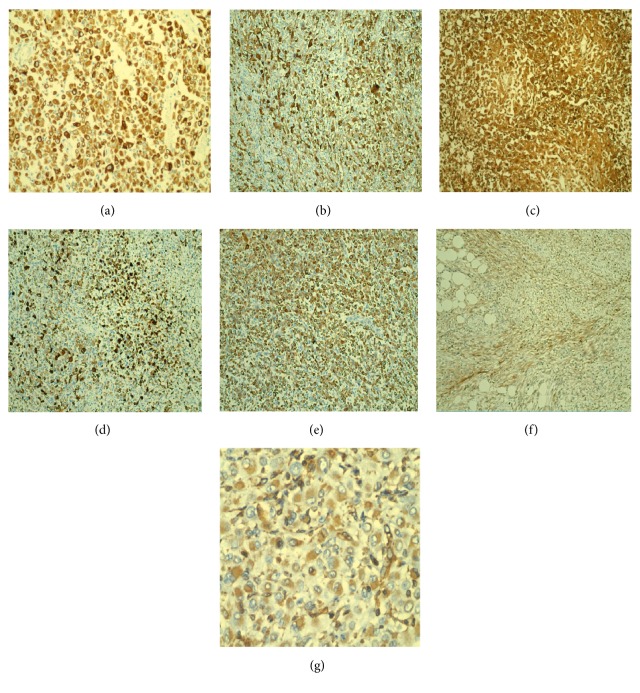
(a) AE1/AE3; (b) Calretinin; (c) CD10; (d) CDK53; (e) CK 5/6; (f) D2-40; (g) Vimentin.

A battery of immunohistochemical markers was performed on viable sections of the tumor. The tumor showed diffuse and strong positivity in all three components for calretinin, CK 5/6, AE1/AE3, D2-40, CD10, CK5D3, vimentin, and focally EMA, which was supportive of a mesothelial phenotype (Figures 4(a)–4(g)). CEA, MOC31, Ber-EP4, HMWCK, and CK19 were all negative, excluding an epithelial phenotype. Markers of hepatocellular differentiation (albumin mRNA by in-situ hybridization, immunohistochemical stains for Arginase and Hep-Par1) were all negative. INI nuclear staining was retained in the rhabdoid areas. RCC, WT-1, PAX-8, S100, HMB45, Desmin, Myogenin, Inhibin, ERG, FLI-1, CD34, and STAT6 were also negative, lending support to a mesothelial phenotype and ruling out a metastatic tumor.

Given this constellation of findings, a diagnosis of localized biphasic malignant peritoneal mesothelioma with rhabdoid features was appropriate. The abdominal wall margin was extensively positive by more than 2 cm. The liver and colonic margins were negative. Over the course of six months, the patient completed three cycles of adjuvant chemotherapy with cisplatin and pemetrexed. Follow-up CT scan showed disease recurrence and progression with multiple liver and peritoneal metastases, as well as a soft tissue mass at the site of ileocolonic anastomosis. The patient additionally received 2 doses of pembrolizumab but had a significant decline in performance status and, due to poor prognosis, was transitioned to hospice.

## 3. Discussion and Review of the Literature

Uncertainty remains as to whether localized malignant mesothelioma is merely a gross variant of diffuse malignant mesothelioma with a similar behavior and clinical course or whether localized malignant peritoneal mesothelioma is distinct from diffuse malignant mesothelioma, sharing with it its mesothelial origin and microscopic features only [2]. Some authors believe that intrahepatic mesotheliomas originate from mesothelial cells of Glisson's capsule which subsequently invade the liver. Others believe that Glisson's capsule consists of collagen fibers, fibroblasts, and small blood vessels and has no mesothelial cells of its own, suggesting that intrahepatic mesotheliomas are simply localized peritoneal malignancies [4].

While asbestos is also the best defined risk factor for malignant peritoneal mesothelioma, the link is weaker than in malignant pleural mesothelioma [5]. Other possible etiologies include radiotherapy exposure, thorotrast use, and erionite exposure. Somatic mutations of BAP1 are observed in 23% of malignant mesotheliomas. Germline mutations in the BAP1 gene predispose patients to malignant mesothelioma, uveal melanoma, and other tumors.

Patients with localized malignant peritoneal mesotheliomas usually present with nonspecific symptoms; pain and weight loss are common, and fever can occur secondary to tumor necrosis [4]. The tumor may also present as an incidental finding on imaging studies done for other reasons. In some cases, laboratory findings may also include increased levels of CYFRA (cytokeratin 19 fragment) and hyaluronic acid in pleural effusions and ascites fluid, increased levels of inflammatory proteins, and thrombocytosis due to the production of interleukin 6 [6]. Anemia can be noted in at least one-third of patients when the hemoglobin level is reported and can be attributed to intralesional hemorrhage. Definitive diagnosis is obtained by tumor resection in most cases [4].

Malignant peritoneal mesotheliomas can be of epithelioid, sarcomatoid, or biphasic types. The presence of rhabdoid cells in localized malignant peritoneal mesotheliomas has been reported by Matsukuma et al. [7]. The differential diagnosis depends on gender, location of tumor, past medical history of the patient, and the histologic type of mesothelioma. Epithelioid malignant peritoneal mesotheliomas should be distinguished from serous papillary carcinoma of the ovary and peritoneum. The differential diagnosis in epithelioid malignant peritoneal mesotheliomas can be divided even further depending on the cytology (clear cell, for example, renal cell carcinoma, squamous cell carcinoma with clear cell features, signet ring, for example, metastatic invasive lobular carcinoma of the breast, metastatic signet ring adenocarcinoma of gastrointestinal origin, deciduoid, for example, pseudotumoral deciduosis, adenoid cystic, for example, female adnexal tumor of probable Wolffian origin, small cell, for example, lymphoma, metastatic small cell carcinoma, rhabdoid, for example, proximal type epithelioid sarcoma, rhabdomyosarcoma, pleomorphic with multinucleated giant cells, for example, metastatic pleomorphic carcinoma of the lung, cells with foamy or vacuolated cytoplasm, or hobnail cells, for example, clear cell carcinoma), and architecture of the cells (tubulopapillary, for example, borderline serous tumors or serous carcinomas of primary peritoneal or Müllerian origin, metastatic papillary thyroid carcinoma arising from a stroma ovarii, metastatic lung adenocarcinoma, and adenomatoid, for example, adenomatoid tumor, acinar, or tubular, for example, metastatic adenocarcinomas or Sertoli-Leydig cell tumors or solid, for example, poorly differentiated carcinoma or lymphoma) of the cells in question. Metastatic mucinous adenocarcinoma and pseudomyxoma peritonei may be confused with epithelioid malignant peritoneal mesothelioma with a myxoid stroma. Sarcomas of the abdominal wall, peritoneum, or intestines enter into the differential diagnosis when dealing with sarcomatoid malignant peritoneal mesotheliomas, including extraintestinal gastrointestinal stromal tumors (GIST) with spindled features; when heterologous (for example, chondrosarcomatous, osteosarcomatous) elements are present in association with the neoplastic spindled cells, metastatic chondrosarcoma and osteosarcoma become diagnostic possibilities. In cases of biphasic malignant peritoneal mesotheliomas in females, carcinosarcomas of Müllerian origin should be excluded with appropriate immunohistochemical studies [8, 9]. Another mimicker of biphasic malignant peritoneal mesothelioma is synovial sarcoma. Tumors that metastasize to the serosal surface and elicit a desmoplastic response can also mimic biphasic malignant peritoneal mesothelioma. Melanoma, sarcomatoid carcinoma, and epithelioid sarcoma should always be ruled out when dealing with spindled or epithelioid neoplastic cells of uncertain origin.

The histological diagnosis of malignant mesothelioma is challenging. To confirm a diagnosis of epithelioid malignant mesothelioma, the current recommendation is to use at least two mesothelial markers (the most useful ones being calretinin, WT-1, cytokeratin 5/6, and D2-40) and two carcinoma markers with greater than 80% sensitivity and specificity (the most useful ones being MOC31, BG8, CEA, and BerEp4). Additional markers would be needed if any of the results are discordant [8]. Studies have shown that among mesothelial markers, calretinin, and WT1 are the most sensitive [10]. In WT-1 negative cases, it is recommended to use at least two mesothelial markers and four other markers based on the differential diagnosis. Sarcomatoid mesotheliomas and the sarcomatoid component of biphasic mesotheliomas may lose immunoreactivity for most markers in the majority of cells; however, calretinin and D2-40 are more likely to remain reactive [8]. The European Respiratory Society/European Society of Thoracic Surgeons (ERS/ESTS) guidelines recommend the use of at least two broad-spectrum cytokeratin antibodies and two markers with negative predictive value for the diagnosis of sarcomatoid mesothelioma [6]. A 55% rate of loss of BAP1 has been described in malignant peritoneal mesotheliomas in a large cohort of cases with 45% of cases showing retention of BAP1, limiting the significance of this marker for the diagnosis of malignant mesothelioma to BAP1 negative cases. Claudin-4 and PAX-8 are very useful markers that are positive in the majority of Müllerian carcinomas with no reported positivity in malignant peritoneal mesotheliomas, and in females can be used to distinguish primary serous carcinoma from epithelioid malignant peritoneal mesothelioma [10].

Most malignant peritoneal mesotheliomas are diffuse and difficult to resect, therefore, the treatment in those cases consists of chemotherapy. This is in contrast to localized peritoneal mesotheliomas, which are amendable to surgical resection [11]. Surgery is, therefore, the mainstay of treatment; however, recurrence occurs a few months after surgery and long-term survival is rarely achieved. Radiation is only feasible for local tumor control and multimodality treatments with chemotherapy can often only achieve partial remission [4].

A detailed search of relevant publications of localized malignant peritoneal mesothelioma was conducted using PubMed and MEDLINE and is summarized in Table 1.

90% of the tumors arose either in the liver or in close proximity to the liver, and 50% of the tumors were epithelioid with the remaining 50% of the tumors comprising both sarcomatoid and biphasic types, of which two cases contained rhabdoid features (one sarcomatoid and one biphasic). 75% of the cases had no prior history of asbestos exposure, including 3 cases with no reported history of asbestos exposure. Based on the follow-up data reported, the clinical course is variable with 5 cases having no recurrence postsurgery, 2 cases resulting in the death of the patient, one case with distal recurrence postoperatively, 2 cases with postoperative disease progression in the form of either nodal, peritoneal, or liver metastases, and 1 case with tumor rupture and subsequent deterioration in the clinical condition of the patient, preventing tumor resection in that case. The tumors appear to affect the older population and have no gender preference.

## 4. Conclusion

Localized malignant peritoneal mesotheliomas are rare tumors that may present clinically as a liver mass and simulate primary hepatic or secondary tumors. They have nonspecific signs and symptoms and need a high index of suspicion and an extensive workup prior to surgery. Not all patients have a history of asbestos exposure. Due to limitations of sampling and the rarity of the tumor, the diagnosis may be difficult to confirm on biopsy or may be overlooked. Definitive diagnosis is obtained by surgical resection in most cases. These patients have a variable clinical course with an unfavorable outcome reported in the majority of cases; however, accumulation of more cases is necessary to characterize the biological behavior and prognosis of these uncommon tumors.

## Figures and Tables

**Table 1:** Literature review and data analysis.

Study	Year	Gender	Age	Location	Histologic Type	Asbestos Exposure	Follow-up
Kottke-Marchan	1989	Female	83	Intrahepatic	Sarcomatoid	Not reported	Unknown

Matsukuma et al.	1996	Male	68	Abdominal wall near liver	Sarcomatoid with rhabdoid cells	No	Died 10 months after surgery

Imura et al.	2002	Male	64	Intrahepatic	Epithelioid	No	Unknown

Sul et al.	2003	Female	55	Liver capsule	Not specified	No	Free of symptoms for 3 months post-surgery

Leonardou et al.	2003	Female	54	Intrahepatic	Epithelioid	Not reported	Unknown

Di Balasi et al.	2004	Female	61	Intrahepatic	Epithelioid	Not reported	Inguinal and peritoneal recurrence

Gutgemann et al.	2006	Male	62	Intrahepatic	Epithelioid	No	Alive and free of disease 36 months post-operatively

Kim et al.	2008	Male	53	Intrahepatic	Biphasic	No	Unknown

Bucholz et al.	2009	Female	62	Intrahepatic	Epithelioid	No	Intraabdominal and intrathoracic lymph node metastases 5, 12 and 20 months post-operatively

Sasaki et al	2009	Male	66	Intrahepatic	Biphasic	Yes	No recurrence or metastasis 6 months post-operatively

Kohno et al.	2012	Male	69	Left anterolateral abdominal wall	Biphasic	Yes	No recurrence over the seven months after the surgery

Inagaki et al.	2013	Female	68	Intrahepatic	Epithelioid	No	Deterioration in general condition of the patient due to hepatic tumor rupture

Dong et al.	2013	Female	50	Intrahepatic	Epithelioid	No	Unknown

Takehara et al.	2014	Male	72	Transverse colon	Biphasic	No	Died 18 months after surgery

Perysinakis et al.	2014	Male	60	Intrahepatic	Epithelioid	No	No recurrence or metastasis 6 months post-operatively

Serter et al.	2015	Female	56	Intrahepatic	Epithelioid	No	Unknown

Serter et al.	2015	Male	66	Intrahepatic	Biphasic	No	Unknown

Ali et al.	2016	Female	41	Intrahepatic	Biphasic	No	Unknown

Ismael et al.	2018	Male	48	Intrahepatic	Epithelioid	No	Unknown

Current case	2018	Female	69	Liver	Biphasic with rhabdoid features	No	Recurrence and progression with multiple liver and peritoneal metastases
