# Corrigendum to “Localized Biphasic Malignant Peritoneal Mesothelioma with Rhabdoid Features Involving the Liver: Case Report and Review of the Literature”

**DOI:** 10.1155/2021/9867879

**Published:** 2021-06-15

**Authors:** Dalal Hassan, Saverio Ligato

**Affiliations:** Department of Pathology and Laboratory Medicine, Hartford Hospital, Hartford, CT, USA

In the article titled “Localized Biphasic Malignant Peritoneal Mesothelioma with Rhabdoid Features Involving the Liver: Case Report and Review of the Literature” [1], the authors have mentioned some grammatical errors. These errors are corrected and highlighted in italic style as below:

In the Abstract:

- “A final diagnosis of localized biphasic malignant peritoneal mesothelioma with rhabdoid features was rendered based on morphology and the *result* of immunohistochemical studies” is corrected to “A final diagnosis of localized biphasic malignant peritoneal mesothelioma with rhabdoid features was rendered based on morphology and the *results* of immunohistochemical studies.”
- “The patient progressed over the course of 6 months despite receiving adjuvant chemotherapy and immunotherapy with metastases and a decline in performance status and was transitioned to *hospice*” is corrected to “The patient progressed over the course of 6 months despite receiving adjuvant chemotherapy and immunotherapy with metastases and a decline in performance status and was transitioned to *hospice care*.”

In Introduction:

“The aim is to increase awareness of localized peritoneal mesothelioma among surgical pathologists, as they can mimic as primary or secondary hepatic tumors clinically and histologically and review the literature with *regards* to prognosis of these uncommon tumors” is corrected to “The aim is to increase awareness of localized peritoneal mesothelioma among surgical pathologists, as they can mimic as primary or secondary hepatic tumors clinically and histologically and review the literature with *regard* to prognosis of these uncommon tumors.”

In Case Presentation:

“The patient additionally received 2 doses of pembrolizumab but had a significant decline in performance status and, due to poor prognosis, was transitioned to *hospice*” is corrected to “The patient additionally received 2 doses of pembrolizumab but had a significant decline in performance status and, due to poor prognosis, was transitioned to *hospice care*.”

In the Figure 3 caption:

“(f) *tumor invading colonic adventitia* (g) *rhabdoid component*” is corrected to “(f) *rhabdoid component and* (g) *tumor invading colonic adventitia*”.

In the Figure 4 caption:

“(d) *CDK53*” is corrected to “(d) *CK5D3*.”

In Discussion and Review of the Literature:

“This is in contrast to localized peritoneal mesotheliomas, which are *amendable* to surgical resection [11]”, is corrected to “This is in contrast to localized peritoneal mesotheliomas, which are *amenable* to surgical resection [11].”
